# No complexity–stability relationship in empirical ecosystems

**DOI:** 10.1038/ncomms12573

**Published:** 2016-08-24

**Authors:** Claire Jacquet, Charlotte Moritz, Lyne Morissette, Pierre Legagneux, François Massol, Philippe Archambault, Dominique Gravel

**Affiliations:** 1 Département de Biologie, Université du Québec à Rimouski, 300 Allée des Ursulines, Quebec, Canada G5L 3A1; 2 Quebec Center for Biodiversity Science, Montréal, Quebec, Canada H3A 1B1; 3 UMR MARBEC, Université de Montpellier, Place Eugène Bataillon, F-34095 Montpellier cedex 05, France; 4 Institut des Sciences de la Mer de Rimouski, Université du Québec à Rimouski, 310 Allée des Ursulines, Quebec, Canada G5L 3A1; 5 Centre de Recherches Insulaires et Observatoire de l'Environnement, EPHE, PSL Research University, UPVD, CNRS, USR 3278 CRIOBE, F-98729 Moorea, French Polynesia; 6 M-Expertise Marine, 10 rue Luce-Drapeau, Sainte-Luce Quebec, Canada G0K1P0; 7 Unité Evolution, Ecologie & Paléontologie (EEP), SPICI group, CNRS UMR 8198, Université Lille 1, Bâtiment SN2, F-59655 Villeneuve d'Ascq cedex, France; 8 Québec-Océan, Département de biologie, Université Laval, Pavillon Alexandre Vachon, 1045, avenue de la médecine, Quebec, QC, Canada G1V 0A6; 9 Département de biologie, Faculté des Sciences, Université de Sherbrooke, 2500 Boulevard Université, Sherbrooke, Quebec, Canada J1K 2R1

## Abstract

Understanding the mechanisms responsible for stability and persistence of ecosystems is one of the greatest challenges in ecology. Robert May showed that, contrary to intuition, complex randomly built ecosystems are less likely to be stable than simpler ones. Few attempts have been tried to test May's prediction empirically, and we still ignore what is the actual complexity–stability relationship in natural ecosystems. Here we perform a stability analysis of 116 quantitative food webs sampled worldwide. We find that classic descriptors of complexity (species richness, connectance and interaction strength) are not associated with stability in empirical food webs. Further analysis reveals that a correlation between the effects of predators on prey and those of prey on predators, combined with a high frequency of weak interactions, stabilize food web dynamics relative to the random expectation. We conclude that empirical food webs have several non-random properties contributing to the absence of a complexity–stability relationship.

## Abstract (web summary)

A long-standing ecological hypothesis is that complexity should decrease stability in food webs. Here, Jacquet and colleagues analyse over 100 real-world food webs and show that complexity does not decrease stability, but that a high frequency of weak species interactions stabilizes complex food webs.

## Graphical abstract

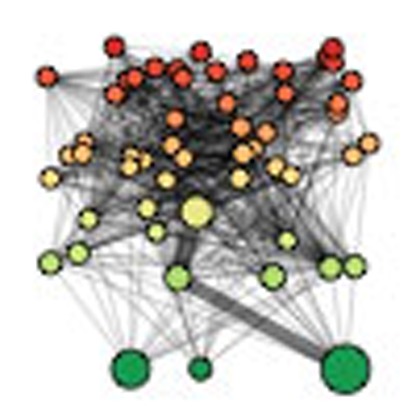

The complexity–stability debate^1^, initiated more than 40 years ago, stems from two apparently conflicting observations. On the one hand, complex ecosystems are ubiquitous in nature, as illustrated by diverse tropical forests, coral reefs or intertidal communities. These observations have inspired ecologists to hypothesize that complexity could stabilize ecosystems^2^,^3^. On the other hand, theory states that complex random systems are less likely to recover from small perturbations than simpler ones^4^,^5^,^6^. This prediction was put forth by Robert May^7^, who studied the relationship between complexity and stability in random ecosystems. Ecosystem complexity was defined as 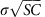 where *S* is species richness, *C* is connectance (the probability that any two species will interact with each other) and *σ* is the s.d. of interaction strength. May^7^ predicted that a system could be stable only if the criterion 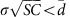 was satisfied, where 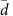 expresses the magnitude of intraspecific competition.

May^7^ analysed the local stability of randomly generated community matrices. A community matrix is obtained from the linearization around a feasible equilibrium of a system of equations describing the dynamics of the community. The entries of a community matrix quantify the impact of a change in abundance of one species on the dynamics of another species. The real part of the dominant eigenvalue of the community matrix indicates the rate at which a system returns to equilibrium (if negative) or moves away from it (if positive) after small perturbations. It does not guarantee stability following large perturbations (global stability), or that the perturbation will not first amplify before vanishing (reactivity)^8^,^9^.

The stability of a random community matrix can be predicted thanks to the generalization of the circular law^10^. This theory states that the distribution of the eigenvalues of a *S* × *S* matrix, whose coefficients are independently sampled from a distribution of mean 0 and variance 1, converges to the uniform distribution in the unit circle in the complex plane, as *S→*∞. The centre of the circle 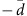 corresponds to the mean of intraspecific interaction terms 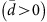, provided that the variance in intraspecific interaction terms is not too large^11^. The radius *R* is related to interspecific interactions and is equal to 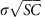 in random ecosystems, that is May's complexity measure. Thus, local stability is determined by the combination of two components; one can increase the stability of a system by (i) moving the centre of the circle to more negative values along the real axis by increasing intraspecific competition or (ii) decreasing the radius of the circle by reducing the complexity of the system (Fig. 1c).

Tang *et al*^12^. proposed that another quantity critically affects the stability of more realistic ecosystems, such as predator–prey communities, namely the correlation between coefficients across the diagonal of the community matrix *ρ*. They subsequently found that the stability criterion for large and random community matrices is 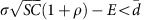 where *E* is the mean of the elements of the community matrix (including zeros). In other words, the correlation between pairs of interactions decreases stability if positive (*ρ*>0) but increases stability if negative (*ρ*<0) with respect to May's case^12^.

Here we attempt to solve the complexity–stability paradox with a local stability analysis of 116 quantitative food webs sampled worldwide from marine, freshwater and terrestrial habitats. This is the largest data set ever used to test May's prediction empirically. The complexity–stability relationship has been previously studied with direct observations of energy flows between species, but on a small number of food webs (from one to seven)^13^,^14^,^15^. Recently, Neutel and Thorne^16^ reported an absence of complexity–stability relationship in 21 soil food webs, while James *et al*^17^. found a weak positive relationship based on 21 food webs from terrestrial and marine habitats. These studies, however, used heterogeneous methodologies, shared several networks, and in several cases, interaction strengths were derived from assumptions rather than from direct observations^18^,^19^.

The studied food webs were all compiled on the same standard methodology to satisfy the Ecopath modelling framework^20^. Ecopath is a trophic model, the most widely used tool for ecosystem-based fisheries management, and has also been used to characterize unexploited terrestrial ecosystems^21^. It relies on a system of linear equations established with the aim of balancing the inflows and the outflows of each compartment^20^,^22^. A large amount of information is included in Ecopath models, such as diet composition, biomass, production and consumption rates of each species, providing an accurate representation of feeding interactions within food webs. Ecopath models provide a unique opportunity to build realistic community matrices with empirical data derived from a standardized protocol. The level of resolution of marine Ecopath models is, however, heterogeneous through food web compartments, with detailed compartments for collected fishes and more aggregated compartments for plankton and invertebrates.

We translated parameters of the Ecopath models into interaction coefficients of the Lotka–Volterra interaction model following the same approach as De Ruiter *et al*^13^. Interaction coefficients from all pairwise interactions of a food web make the interaction matrix **A**=[*α*_*ij*_]. Because of the equilibrium assumption of Ecopath models, a community matrix **C** can be constructed for each food web by multiplying the interaction matrix **A** with species biomass (Fig. 1b).

We measured food web stability using the real part of the dominant eigenvalue of the community matrix **C** to be directly comparable to May's approach. The diagonal elements of the community matrices were set to 0 to focus on the effect of interspecific interactions on stability. Note that *Re*(*λ*_max_) will be positive, since *R*>0 (Fig. 1c). This method is comparable to other studies that calculated stability by assessing the level of intraspecific interaction needed for all eigenvalues in a community matrix to have negative real parts^14^,^16^,^19^.

We show that complexity is not related to stability in empirical ecosystems. We find that the intrinsic energetic organization of food webs creates a high frequency of weak interactions and a correlation between pairs of interactions. These non-random properties are highly stabilizing and contribute to the absence of a complexity–stability relationship.

## Results

### Complexity–stability relationship in empirical ecosystems

We first investigated the relationship between stability and classic descriptors of ecosystem complexity^23^, that is, species richness *S*, connectance *C* and s.d. of interaction strengths σ. We observed no relationship between food web stability and species richness, neither with connectance nor with s.d. of interaction strength (Fig. 2). Further analyses revealed that this result was robust to the variability of sampling intensity among the 116 food webs and to uncertainty related to Ecopath parameter estimates (Methods section, Supplementary Figs 1,2 and 3). We neither found significant complexity–stability relationship using the stability criterion derived by Tang *et al*^12^. that integrates correlation between pairs of interactions and mean of interaction strengths (Supplementary Fig. 4). The absence of a complexity–stability relationship in empirical food webs demonstrates that the random matrices studied by May^7^ to derive stability criteria deviate significantly from empirical systems. As May^7^ stated in the re-edition of his book, his theory provides the baseline against which we should compare empirical systems and find the non-random features stabilizing them. We therefore investigated further the mechanisms preventing the negative relationship between complexity and stability to occur.

### Correlation between complexity parameters

May's stability criterion 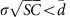 indirectly predicts that for complex systems to persist, interaction strength should be weaker in species-rich and highly connected systems^7^,^24^. In other words, complex ecosystems could persist in nature provided that *S*, *C* and *σ* are not independent. In the same way, the inequality derived by Tang *et al*.^12^, 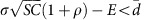, predicts that *ρ* and 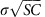 should be correlated in feasible ecosystems. We therefore hypothesized that, contrary to randomly built ecosystems, parameters describing complexity are not independent in nature. We found that the s.d. of interaction strength *σ* across the 116 food webs was negatively correlated to the product of species richness and connectance 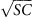 (Fig. 3a) and contrary to expectations, we observed a slightly positive correlation between *ρ* and 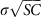 (Fig. 3b). The correlation between σ and 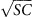 decreased the overall complexity and confirmed the existence of feasibility constraints on communities. However, we still observed higher values of *σ* than predicted by May's stability criterion and this observation did not explain the absence of complexity–stability relationship in empirical systems.

### Non-random properties of empirical community matrices

Random matrix theory supposes that interaction coefficients are independent and identically distributed in the community matrix. However, many studies on the complexity-stability relationship suggest that real ecosystems have non-random structural properties promoting their stability despite their complexity^23^. We focused on four non-random properties observed in our empirical community matrices, and then investigated their contribution to stability with randomization tests.

**Figure 1:**
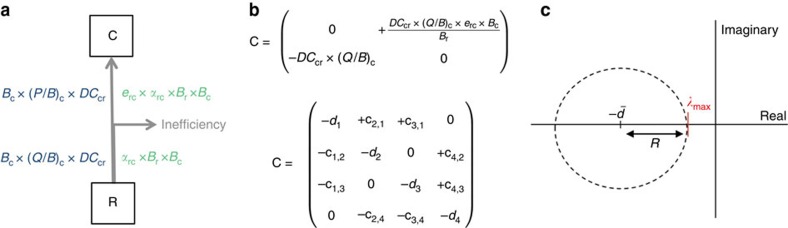
Method summary. (**a**) Equivalence between Ecopath and Lotka–Volterra models: simplified diagram of trophic flows between one consumer *C* and one resource *R* parameterized with Ecopath model (in blue) and Lotka–Volterra model (in green). *B* is biomass (t km^−2^), (*P/B*)_*c*_ and (*Q/B*)_*c*_ are the production/biomass and consumption/biomass ratios of *C* respectively (per year), *DC*_*cr*_ is the proportion of resource *R* in the diet of consumer *C*, *e*_*rc*_ expresses the efficiency of a consumer to convert resource energy into biomass with *e*_*rc*_*=*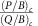. (**b**) Community matrix construction: derivation of community matrix elements for the simplified food web presented in diagram A, and an example of community matrix structure observed in real food webs. (**c**) Measure of stability: the eigenvalues of a large community matrix are uniformly distributed on a circle on the complex plane (axes cross at the origin). On the real axis, the dominant eigenvalue *Re*(*λ*_max_)=*R−*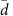, where the centre of the circle 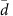 is equal to the mean of intraspecific interaction terms 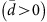, and the radius *R* is related to interspecific interaction terms (that is, off-diagonal elements of the community matrix) and is equal to 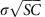 in random matrices. For predator-prey communities, the eigenvalues are uniformly distributed on an ellipse with a horizontal half axis 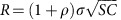 (ref. 11).

**Figure 2:**
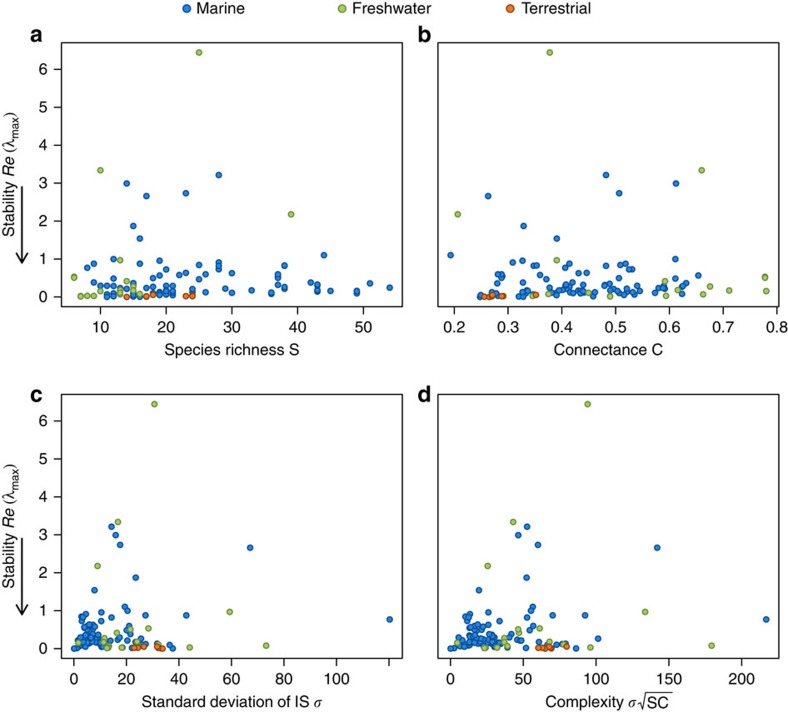
Food web stability related to complexity parameters in 116 food webs. (**a**) Number of species *S* (linear regression: *P*=0.97, *R*^2^<10^−5^), (**b**) Connectance *C*=(*L/S*^2^) where *L* is the number of links *(P*=0.98, *R*^2^<10^−6^), (**c**) Standard deviation of interaction strengths σ (*P*=0.1, *R*^2^=0.02), (**d**) May's complexity measure 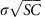 (*P*=0.02, *R*^2^=0.04). Stability is measured as *Re*(*λ*_max_) for marine (blue), freshwater (green) and terrestrial ecosystems (orange). Food webs with eigenvalues close to zero are the most stable. All quantities are dimensionless.

(i) Pyramidal structure of interaction strength^13^,^17^,^18^: we found that interaction strength was related to trophic level, the occurrence of strong interactions being more likely at low trophic levels. Species biomass distribution affected the mean and variance of row *i*, since *c*_*ji*_=*a*_*ij*_ × *B*_*j*_. Consequently, rows had different means and variance, a feature we call row structure. We hypothesized that food webs without this row structure are less stable than real food webs. (ii) Interaction strength topology^14^,^15^,^18^: trophic structure determines the position and the direction of interaction strength (that is, ‘who eats whom'), and creates a non-random topology of interaction strengths. We hypothesized that food webs with a random topological structure are less stable than real food webs. (iii) Correlation between pairs of predator-prey interactions^12^,^25^,^26^: we found a correlation between pairs of interaction strengths *c*_*ij*_ and *c*_*ji*_ in community matrices, since *c*_*ji*_=(*−c*_*ij*_ × *e*_*ij*_ × *B*_*j*_)/*B*_*i*_ (Fig. 1b). We therefore hypothesized that food webs with an empirical topological structure, but with a null correlation between pairs of interaction strengths, are less stable than real food webs. (iv) Interaction strength frequency distribution: in agreement with previous studies^13^,^27^,^28^,^29^, we observed a leptokurtic distribution of interaction strengths (high proportion of weak interactions). Consequently, we hypothesized that food webs with a highly peaked and long tailed distribution of interaction strengths are more stable than flatter distributions, such as the normal distribution.

### Randomization tests

We performed eight randomization tests to remove one or several properties of natural food webs and computed stability of the permuted community matrices (called H1–H8 at Table 1, see Methods section for details). We used this method to determine whether these properties had a significant effect on the distribution of eigenvalues across the 116 food webs, and their impact on the complexity–stability relationship. Randomization tests removed some non-random features of empirically built community matrices, generating matrices more similar to the ones expected under the random matrix theory, in which elements are drawn from a standardized distribution.

The distribution of eigenvalues of the permuted food webs was compared to stability of the original food webs. We found that each of the four structural properties enhanced food web stability (Fig. 4a). The removal of the empirical distribution of interaction strengths (with many weak interactions, H4) had the strongest impact on stability, followed by the removal of correlation between pairs of interactions (H3). Note that in all the randomization tests, the pyramidal structure of interaction strength was removed. Stability decreased when only this property was removed, keeping empirical topology, pairwise correlation and interaction strength distribution (H1). The randomization of interaction strength topology (H2) was also destabilizing, but to a very lesser extent compared with others non-random properties (Fig. 4a).

**Figure 3:**
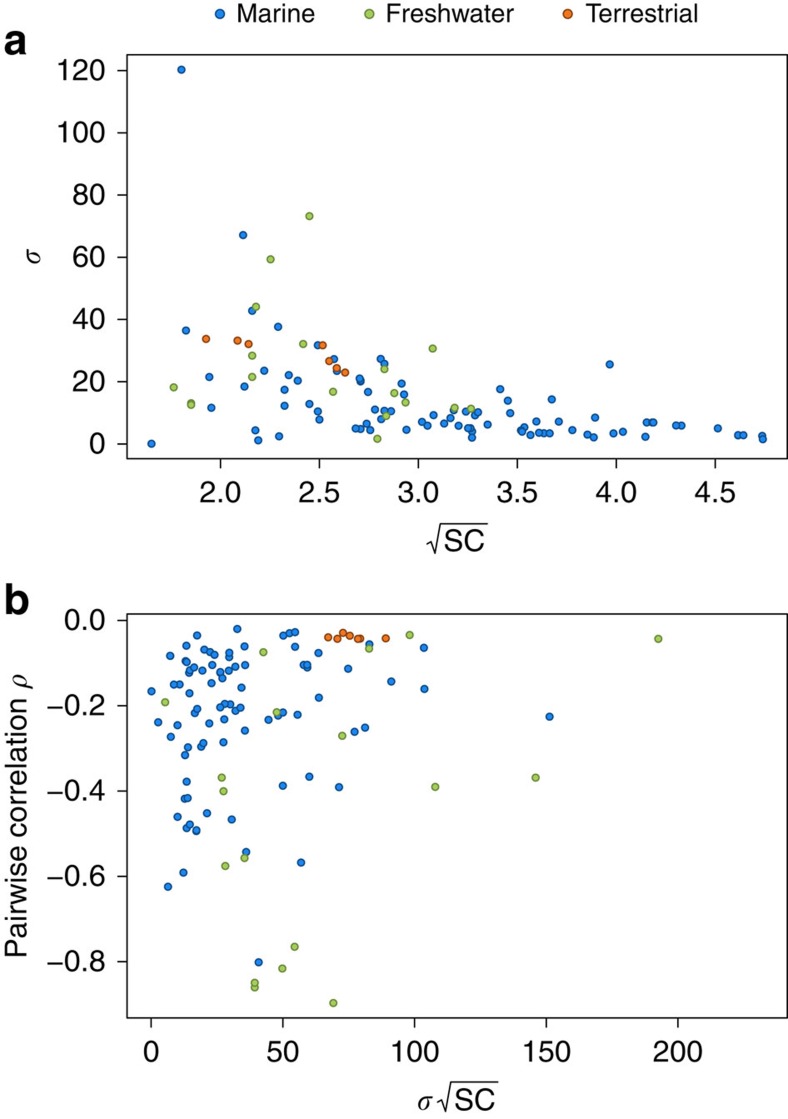
Correlation between complexity parameters in real food webs. (**a**) σ is the s.d. of interaction strengths, *S* the number of species and *C* the connectance. The product 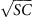 was negatively correlated to σ (Spearman's rank correlation (*P*<10^−13^, *r*=−0.64). (**b**) 
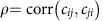
 is the correlation between pairwise interactions. The product 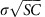 was positively correlated to *ρ* (*P*=0.02, *r*=0.22).

**Figure 4:**
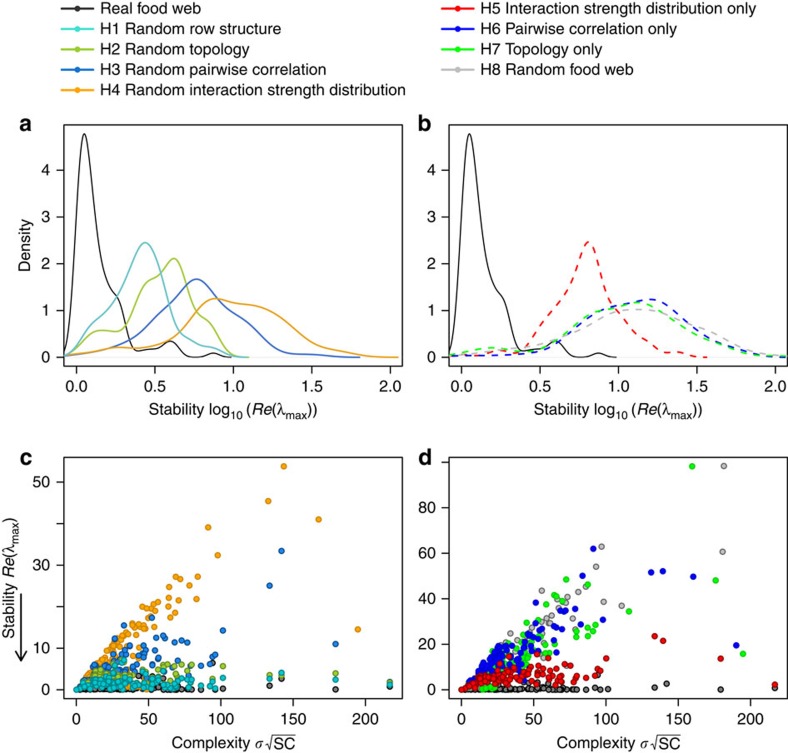
Complexity–stability relationship in empirical and permuted food webs. Frequency distributions of eigenvalues of real and permuted food webs : (**a**) permutation tests H1 to H4, (**b**) permutation tests H5 to H8. Eigenvalues are on a logarithmic scale and dimensionless. Permutation tests were carried out 1,000 times for each food web. Eigenvalue distributions were smoothed using a kernel density estimate of 0.28. (**c**) Stability of real and permuted food webs related to complexity (permutation tests H1–H4). Stability is measured as *Re(λ*_*max*_) and 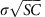 corresponds to complexity. Statistics of the linear regression between complexity and stability: real food webs (slope=0.005, *P*=0.02, *R*^2^=0.04), H1: random row structure (slope=0.003, *P*=0.38, *R*^2^=0.002), H2: random topology (slope=0.006, *P*=0.2, *R*^2^=0.006), H3: random pairwise correlation (slope=0.06, *P*=10^−7^, *R*^2^=0.2), H4: random interaction strength distribution (slope=0.24, *P*<10^−16^, *R*^2^=0.65). (**d**) Stability of permuted food webs related to complexity (permutation tests H5–H8). Statistics of the linear regression between complexity and stability: H5: empirical distribution of interaction strengths only (slope=0.06, *P*=10^−9^, *R*^2^=0.25). H6: pairwise correlation only (slope=0.3, *P*<10^−16^, *R*^2^=0.63). H7: topology only (slope=0.32, *P*<10^−16^, *R*^2^=0.6). H8: random food webs (slope=0.33, *P*<10^−16^, *R*^2^=0.66).

Randomization tests resulted in some cases in a negative complexity–stability relationship, although weaker than one should expect from the random matrix theory. Even if randomized matrices conserved the same S, C and σ^2^ as original ones (and thus their correlation, presented in Fig. 3a), we found a negative complexity–stability relationship when we normalized interaction strength distribution (H4, linear regression: *P*<10^−16^, *R*^2^=0.64) and removed correlation between pairs of interactions (H3, linear regression: *P*=10^−7^, *R*^2^=0.2, Fig. 4c). The removal of the pyramidal structure of interaction strengths and the topology found in empirical ecosystems did not affect the relationship between complexity and stability (linear regressions, H1: *P*=0.38, *R*^2^=0.002, H2: *P*=0.2, *R*^2^=0.006, Fig. 4c).

All food web properties contributed to stability, but clearly, the leptokurtic distribution of interaction strength had the strongest impact on the complexity–stability relationship. We found a significant negative relationship between stability and complexity when we removed this property (H4, Fig. 4c). Conversely, when we only kept the empirical distribution of interaction strengths (H5), the slope of the complexity–stability relationship was significantly flatter than in the random case (H8). Topology of interaction strengths (H7) or pairwise correlation (H6) alone did not significantly affect the complexity–stability relationship (Fig. 4d).

We conclude that May's stability criterion does not apply to empirical ecosystems because of their structure, which has several stabilizing non-random properties. First, the high frequency of weak interactions balanced the destabilizing effect of complexity (H4). Interestingly, we observed a strong positive correlation between the kurtosis *κ* (index of the peakedness of the interaction strength distribution) and species richness in real food webs (Supplementary Fig. 5). Thus the probability of having many weak interactions increased with species richness. The negative correlation between pairs of interaction strengths *c*_*ij*_ and *c*_*ji*_ is also a strong stabilizing property of empirical community matrices (H3). Finally, the non-random topology of interaction strengths (H2) was also stabilizing, as suggested by previous studies^13^,^14^,^16^,^18^.

## Discussion

The relevance of local stability analysis to study real ecosystems may be questioned. More general and realistic definitions of stability have been introduced during the complexity–stability debate, such as persistence, variability, resilience or resistance^30^. Indeed, local stability analysis only tests the impact of small perturbations on ecological dynamics, and may not apply to large and/or cumulative perturbations typical of most empirical studies. It neither considers the covariance among species and thus the stability of the aggregated properties of the community^31^. However, it allows the use of analytically tractable community matrices, and thus the investigation of May's complexity–stability relationship on real ecosystems.

Our study yields new insight on the complexity–stability debate. Random matrix theory cannot predict the stability of real ecosystems because interaction strengths are not independent and identically distributed in empirically derived community matrices. Trophic structure creates a negative correlation between pairs of interactions and a non-random distribution of interaction strengths, with many weak interactions and few strong ones at the bottom of the food webs. The likely explanation for the strong effect of the leptokurtic distribution of interaction strengths is the size of the community matrices we investigated. Random matrix theory is performed in the limit of infinitely large matrices and all distributions are expected to converge in systems of several hundreds of species^11^. The community matrices we investigated had between 6 and 54 species. A detailed investigation of some community matrices revealed that small modules (two to five species) were often responsible for extreme eigenvalues. These modules could drive strong negative or positive feedbacks^16^ and thus dominate the dynamics of the entire system. Random matrix theory could provide a sufficient approximation for large ecosystems, but needs to be refined for smaller and realistic food webs such as the ones we investigated.

The study of small community matrices might require a different theoretical framework. For instance, Neutel and Thorne^16^ showed that the stability of a dynamical system could be predicted from the analysis of feedback loops. However, this approach requires knowledge of all of the elements of the community matrix and does not provide a statement about the expected relationship between *S*, *C*, *σ* and the occurrence of feedback loops. Such a theory would be needed to make quantitative predictions about the stability of a system with estimates of only few state variables.

Our food web dataset provided a great opportunity to study the effect of interspecific interactions on the relationship between complexity and stability and to demonstrate the existence of a negative correlation between *S*, *C* and *σ* in empirical ecosystems. We had, however, no information about the strength of intraspecific interactions, which is a strong stabilizing mechanism. Our analysis thus focused on the radius of the distribution of eigenvalues in the complex plane, ignoring the location of the centre (Fig. 1c). It is possible that the absence of relationship between complexity and stability results from a positive correlation between the strength of intraspecific interactions 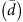 and complexity 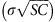. Here we hypothesized that the food webs we studied were mainly top-down controlled, and that the strength of intraspecific interactions was negligible in comparison to interspecific interactions. Nonetheless, we evaluated the sensitivity of our findings to the addition of intraspecific interaction terms proportional to species equilibrium biomass, since *c*_*ii*_=*α*_*ii*_ × *B*_*i*_. In agreement with random matrix theory and previous studies^11^,^14^,^19^, the addition of intraspecific interactions was stabilizing, but had no effect on the correlation between complexity and stability (Methods section, Supplementary Fig. 6). Our results emphasize that further empirical investigations should better consider the relationship between ecosystem complexity and density dependence.

The analysis of empirically derived community matrices, combined with the observation of a complexity–stability relationship when their non-random structural properties were removed, demonstrates that the properties captured by Ecopath models contribute to the stability of complex food webs. Further empirical investigations are necessary to better approach real ecosystems and to study the stabilizing effect of the properties ignored or poorly described in Ecopath models, such as species age structure, energy flows from detrital pool or external inputs.

We showed that complexity is not related to stability in empirical ecosystems, a question that has stimulated ecological research for four decades. We found that the intrinsic energetic organization of food webs is highly stabilizing and allows complex ecosystems to recover from perturbations. Coexistence also constrains the feasibility of ecosystems, imposing a non-random structure of interactions and a correlation between *S*, *C* and *σ* that decreases the overall complexity^24^. The non-random structure of food webs occurs from the successive addition of consumers having an increasingly large diet, which causes a growing frequency of weak interactions. The complexity–stability debate has contributed to the development of productive research that have pointed out the key role of the structural properties of real ecosystems.

## Methods

### Ecopath modelling framework

We compiled 116 Ecopath food web models from published studies (Supplementary Table 1). Ecopath provides a quantitative overview of how species interact in a food web. Species sharing the same prey and predators and having similar physiological characteristics are aggregated in trophic species. The dynamics of each species *i* is described by the difference between biomass production and biomass losses due to harvesting, predation or other unspecified sources. It can be expressed as:


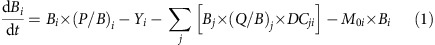


where *B*_*i*_ (t km^−2^) and (*P/B*)_*i*_ (per year) are biomass and production/biomass ratio of species *i*, respectively, *Y*_*i*_ (t km^−2^ per year) corresponds to fishery yields, (*Q/B*)_*j*_ (per year) is consumption/biomass ratio of predator *j* and *DC*_*ji*_ is the proportion of species *i* in the diet of predator *j*. Other mortality sources, *M*_*0i*_ (per year), can be expressed as (*1−EE*_*i*_) × (*P/B*)_*i*_, where *EE*_*i*_ is called the ecotrophic efficiency of *i*, corresponding to the fraction of the production that is used in the food web. The Ecopath model assumes mass-balance, meaning that all species biomass are at equilibrium (d*B*_*i*_/d*t*=*0*).

Input parameters (that is, biomass, production/biomass and consumption/biomass ratios, fishery yields, and diet composition) can have different origins: field sampling (for example, trawl survey), derived from similar Ecopath models, or known empirical relationships. Ecopath with Ecosim software includes routines that estimate missing parameters based on the mass-balance hypothesis and the generalized inverse method for a system of n linear equations and m unknowns (see Christensen *et al*.^22^, p. 12–15). In general, the biomasses, production/biomass and consumption/biomass ratios are entered for all groups to estimate ecotrophic efficiency, which is difficult to measure in the field^32^. The Ecoranger module, also included in Ecopath with Ecosim software, can be used to explore the effect of uncertainty in input data on estimated parameters. This module calculates probability distributions of output parameters based on the confidence intervals of input parameters specified by the users^32^. Full details of the Ecopath modelling approach and the Ecopath with Ecosim software can be obtained from www.ecopath.org.

### Parameterization of Lotka–Volterra interaction coefficients

We used the method from De Ruiter *et al*^13^. to derive the community matrices from Ecopath models (Fig. 1a): assuming direct dependence of feeding rates on predator population density, we calculated the per capita effect of predator *j* on the growth rate of prey *i* as *α*_*ij*_=*−*((*Q/B*)_j_ × *DC*_*ji*_)/*B*_*i*_. Effects of prey on their predator are defined as predator growth resulting from this predation. Consequently, effect of the prey *i* on the predator *j* is related to effect of the predator on the prey according to: *α*_*ji*_=*−e*_*ij*_ × *α*_*ij*_, where *B* is biomass, *DC*_*ji*_ is the proportion of species *i* in the diet of predator *j*, *e*_*ij*_ is the efficiency with which *j* converts food into biomass, from feeding on *i*: *e*_*ij*_=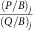 and (*P/B*)_*j*_ and (*Q/B*)_*j*_ are predator production/biomass and consumption/biomass ratios respectively. We obtained the following Lotka–Volterra interaction equation:


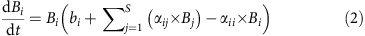


where *b*_*i*_ is the intrinsic growth rate (that is, the intrinsic rate of increase for autotrophs, and natural mortality and losses for heterotrophs), *B*_*i*_ and *B*_*j*_ are, respectively, biomass of species *i* and *j*, interaction strength *α*_*ij*_ corresponds to the per capita effect of species *j* on the growth rate of species *i* and *α*_*ii*_ represents the per capita self limitation of species *i*. Assuming mass-balance, we obtain the following expression for intrinsic growth rate: 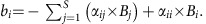


### Correlation between pairs of interactions

Pairwise correlation was calculated using the formula from Tang *et al*.^12^: 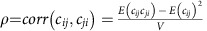 where *E*(*c*_*ij*_) is the mean of the off-diagonal elements *c*_*ij*_ of the community matrix, their variance is *V* and *E*(*c*_*ij*_*c*_*ji*_) is the mean of the products of the pairs *c*_*ij*_*c*_*ji*_.

### Randomization tests

Reported dominant eigenvalues of randomized food webs corresponded to the mean of 1,000 replicates. All permutation tests conserved *S*, *C* and *σ*. To randomize the pyramidal structure of interaction strengths (H1), we swapped pairs of predator-prey interactions (the pair *−c*_*ij*_*/+c*_*ji*_ was replaced by the pair *−c*_*kl*_*/+c*_*lk*_). This permutation only changed row structure (mean and variance) and did not change topology, frequency distribution of interaction strengths nor correlation between pairs of interactions. To randomize interaction strength topology (H2), we swapped the element of the community matrix *c*_*ij*_ with the element *c*_*ji*_. This permutation only removed food web topology and did not change the frequency distribution of interaction strengths or pairwise correlation. To remove correlation between pairs of predator-prey interactions (H3), we permuted off-diagonal elements of the community matrix, keeping the topological structure and the frequency distribution of interaction strengths. Positive and negative terms were permuted separately to keep identical averages of positive and negative interactions. For randomization of interaction strength distribution (H4), we created a random community matrix in which off-diagonal elements were picked from a bivariate normal distribution *N*_*2*_*(μ, Σ)* where the mean vector *μ* is composed of the mean of positive *(μ+)* and the mean of negative *(μ−)* terms, and *Σ* is the covariance matrix between positive and negative terms of the original community matrix. Original pairs of positive/negative terms were replaced by positive/negative terms from the bivariate normal distribution. For large random community matrices, the correlation between pairwise interactions is expected to be identical to the original community matrix. Randomization test H5 only kept frequency distribution of interaction strengths. This test is a combination of permutation H2, that randomizes the topology of interaction strengths, and permutation H3, that removes pairwise correlation. Randomization test H6 only kept pairwise correlation; this test is a combination of permutation H2 and randomization H4, that creates a random community matrix in which off-diagonal elements are picked from a bivariate normal distribution. Randomization test H7 only kept the topology of interaction strengths, which is a combination of tests H3 and H4. Randomization test H8 created community matrices in which elements were identically and independently distributed, that is food webs with a random topology, a normal distribution of interaction strengths and no correlation between pairs of interactions. This test corresponds to test H2, that randomizes the topology of the community matrix, combined to a randomization that creates a community matrix in which positive and negative off-diagonal elements are picked from a normal distribution *N*(*μ*_*+*_*,σ*^2^_*+*_) and *N*(*μ*_*−*_*,σ*^2^_*−*_), where *μ*_*+*_ and *μ*_*−*_ are the mean and *σ*^2^_*+*_ and *σ*^2^_*−*_ the variance of positive/negative elements of the original community matrix.

### Parameter uncertainty

We investigated the impact of parameter uncertainty on our findings. In the section ‘Interspecific interaction terms of the community matrix', we evaluated the sensitivity of our results to variability in interspecific interaction terms. The parameters used to build empirical community matrices come from Ecopath data and each of them bears some uncertainty^22^. Consequently, we tested whether the introduction of variability in input parameters could bias the complexity–stability relationship.

In the section ‘Intraspecific interaction terms of the community matrix', we evaluated the robustness of our results to the addition of density dependence. Because Ecopath models depict exclusively trophic interactions between species, we had no empirical information about the strength of intraspecific interactions and we decided not to model density dependence in the Lotka–Volterra model. Our method is comparable to other studies that calculated stability by assessing the level of intraspecific interaction needed for all eigenvalues in a community matrix to have negative real parts (diagonal dominance)^6^,^16^,^19^. These studies assumed that all diagonal elements *c*_*ii*_ of the community matrix are the same. However, to obtain the community matrix, the interaction matrix **A** is multiplied by species biomass, which means that diagonal elements are non-constant: *c*_*ii*_=α_*ii*_ × *B*_*i*_. We therefore evaluated the robustness of our results to the addition of diagonal elements structured by species biomass.

Finally, in the section ‘Food web resolution', we assessed the impact of food web resolution level on the complexity–stability relationship. Ecopath model is mainly used for ecosystem-based fisheries management and the level of resolution of several food webs is not homogeneous through all ecological compartments. Harvested fishes are generally resolved at the species level, while species at the bottom of the food web, such as plankton and invertebrates, are highly aggregated. We therefore analysed the complexity–stability relationship on a subset of the best resolved Ecopath food webs.

Overall, we found the same qualitative results than our main study. We conclude that our findings are robust to (i) input parameter uncertainty, (ii) addition of non-zero diagonal elements in community matrices and (iii) differences in food web resolution level.

### Interspecific interaction terms of the community matrix

We ran sensitivity analyses to determine how uncertainties in parameter estimates could affect the results of the study. For each input parameter, we tested if uncertainty biases (that is, overestimates or underestimates) food web stability and the variables determining complexity, and if our findings are qualitatively affected by this bias.

We used the following parameters from Ecopath data to determine the interspecific terms of a community matrix: (i) biomass *B*, (ii) consumption/biomass ratio (*Q*/*B*), (iii) production/biomass ratio (*P*/*B*) and (iv) diet composition *DC*. Uncertainty in these parameters could influence our results through the dominant eigenvalue *Re*(λ_max_), through the standard deviation of interaction strength σ (related to May's complexity criterion 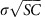), or through the pairwise correlation ρ (related to Tang's complexity criterion 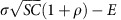).

We used a resampling procedure to evaluate the sensitivity of our results to parameter uncertainty. For each of the 116 Ecopath models, we proceeded as follows: we resampled each parameter, *B*, (*Q*/*B*) and (*P*/*B*), 1,000 times from a normal distribution *N*(*μ*,σ) with *μ*=*X*_*i*_, σ=*X*_*i*_/10 (corresponding to a *CV*=10%) and *X*_*i*_ is the reported value of parameter *X* for species *i*. We chose a *CV* of 10% because higher values could lead to negative *P/B*. We built a matrix of diet composition in which predators have no prey preferences (that is, they are opportunistic feeders, attacking prey in proportion to their availability). The proportion of prey *i* in the diet of predator *j* corresponds to the ratio between biomass of *i* and total biomass of all *j*'s prey species.

(i) Biomass: for each of the 116 Ecopath models, 1,000 community matrices were built from the resampled values of *B*. Diet composition, production/biomass and consumption/biomass ratios were kept constant and corresponded to the values reported in Ecopath data.

(ii) Consumption/biomass ratio: for each of the 116 Ecopath models, 1,000 community matrices were built from the resampled values of *Q*/*B*. Biomass, diet composition and production/biomass ratio corresponded to the values reported in Ecopath data.

(iii) Production/biomass ratio: for each of the 116 Ecopath models, 1,000 community matrices were built from the resampled values of *P*/*B*. Biomass, diet composition and consumption/biomass ratio corresponded to the values reported in Ecopath data.

(iv) Diet composition: for each of the 116 Ecopath models, a community matrix was built using the matrix of diet composition in which predators have no prey preferences. Biomass, production/biomass and consumption/biomass ratios corresponded to the values reported in Ecopath data.

We calculated the dominant eigenvalue *Re*(λ_max_), the standard deviation of interaction strengths *σ* and the correlation between pairwise interactions ρ of these community matrices and compared their values to the ones found in the original community matrices (Supplementary Fig. 2). We found that uncertainty in the estimation of *B*, *P*/*B* and *Q*/*B* had no effect on food web stability or the variables determining complexity. The absence of diet preferences was destabilizing and also decreased s.d. of interaction strength. However, we found that the deviation between the original leading eigenvalues and the ones obtained after the addition of variability in input parameters was not correlated to complexity (Supplementary Fig. 3). The complexity–stability relationship would have been biased if, for instance, the addition of variability in the biomass estimates would have a more profound impact on the leading eigenvalue of highly complex webs than the one of simpler food webs. In agreement with Barabas *et al*^33^. these results demonstrate that our findings are robust to the addition of variability in interspecific interaction terms of the community matrices.

### Intraspecific interaction terms of the community matrix

The diagonal elements *c*_*ii*_ of the community matrix express the strength of density dependence, which is highly stabilizing as it moves the dominant eigenvalue to more negative values: *Re*(λ_*max*_)=*R*−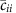 , where the radius of the unit circle *R* corresponds to 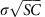 in random matrices and 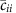 is the mean of diagonal elements (Introduction section and Fig. 1c). Our aim was not to assess the local stability of empirical food webs but to investigate the relationship between stability and complexity using realistic community matrices on a large gradient of complexity. In the main text, we therefore set all the diagonal elements to 0 to focus on the effect of interspecific interactions on stability. Our method is comparable to other studies that calculated stability by assessing the level of intraspecific interaction needed for all eigenvalues in a community matrix to have negative real parts (diagonal dominance)^6^,^16^,^19^. These studies assumed that all diagonal elements *c*_*ii*_ of the community matrix are the same. However, to obtain the community matrix, the interaction matrix **A** is multiplied by species biomass and the diagonal of an empirically derived community matrix should be structured by species biomass, as *c*_*ii*_=α_*ii*_ × Bi*. Here we investigated how a non-constant diagonal, in which elements *c*_*ii*_ are proportional to species biomass, affects the dominant eigenvalues reported in our analysis. We compared the dominant eigenvalues of community matrices with α_*ii*_=0 (original food webs) and α_*ii*_=0.01 or 0.1 (Supplementary Fig. 6). We found that the addition of the intraspecific interaction terms was stabilizing, but had no effect on the absence of complexity–stability relationship.

### Food web resolution

Ecopath is mainly used for ecosystem-based fisheries management. Consequently, the structure of food webs parameterized with Ecopath is often biased, with detailed compartments for harvested fishes and more aggregated compartments for species at the bottom of the food web (that is, plankton and invertebrates). Food web resolution influences the estimation of species richness, connectance and interaction strength. To assess the robustness of our analysis, we investigated the complexity–stability relationship on a subset of the best resolved Ecopath models. We measured the amount of aggregation of each model, based on the criterion that groups with taxonomic name were more resolved than groups with trophic function names. We defined four resolution levels and qualified one level for each species with the following indices: taxonomic species (that is, greenland turbot, index=1), family/class (that is, whales, gadoids; index=0.7), trophic function (that is, small demersal fish; index=0.4) and general name (that is, benthos, fish; index=0.1). Resolution indices *RI* of Ecopath models correspond to the mean resolution index of species within each food web and are listed in Supplementary Table 1. We studied the complexity–stability relationship on a subset of the 37 best resolved models (with *RI*≥0.7) and found results similar to the overall analysis (Supplementary Fig. 1).

### Data availability

The data that support the findings of this study are available from the corresponding author on request.

## Additional information

**How to cite this article:** Jacquet, C. *et al*. No complexity–stability relationship in empirical ecosystems. *Nat. Commun.* 7:12573 doi: 10.1038/ncomms12573 (2016).

## Supplementary Material

Supplementary Information Supplementary Figures 1-6, Supplementary Table 1 and Supplementary References

## Figures and Tables

**Table 1:** Properties conserved by each randomization test (indicated by a ✓).

**Hypothesis**	**Row structure**	**Topology**	**Pairwise correlation**	**Frequency distribution**
H1	×	✓	✓	✓
H2	×	×	✓	✓
H3	×	✓	×	✓
H4	×	✓	✓	×
H5	×	×	×	✓
H6	×	×	✓	×
H7	×	✓	×	×
H8	×	×	×	×

The column ‘row structure' specifies whether the pyramidal structure of interaction strength is conserved or not in randomization tests H1–H8. Similarly, ‘topology' corresponds to interaction strength topology (‘who eats whom'), ‘pairwise correlation' corresponds to the correlation between pairs of predator–prey interactions and ‘frequency distribution' corresponds to the leptokurtic distribution of interaction strengths (high proportion of weak interactions).
