# Supplementary material for: No complexity–stability relationship in empirical ecosystems
Source: Nat Commun. 2016 Aug 24;7:12573. doi: 10.1038/ncomms12573 (PMC4999500; doi:10.1038/ncomms12573)
Supplement: Supplementary Information — Supplementary Figures 1-6, Supplementary Table 1 and Supplementary References [file ncomms12573-s1.pdf]

Supplementary Figures

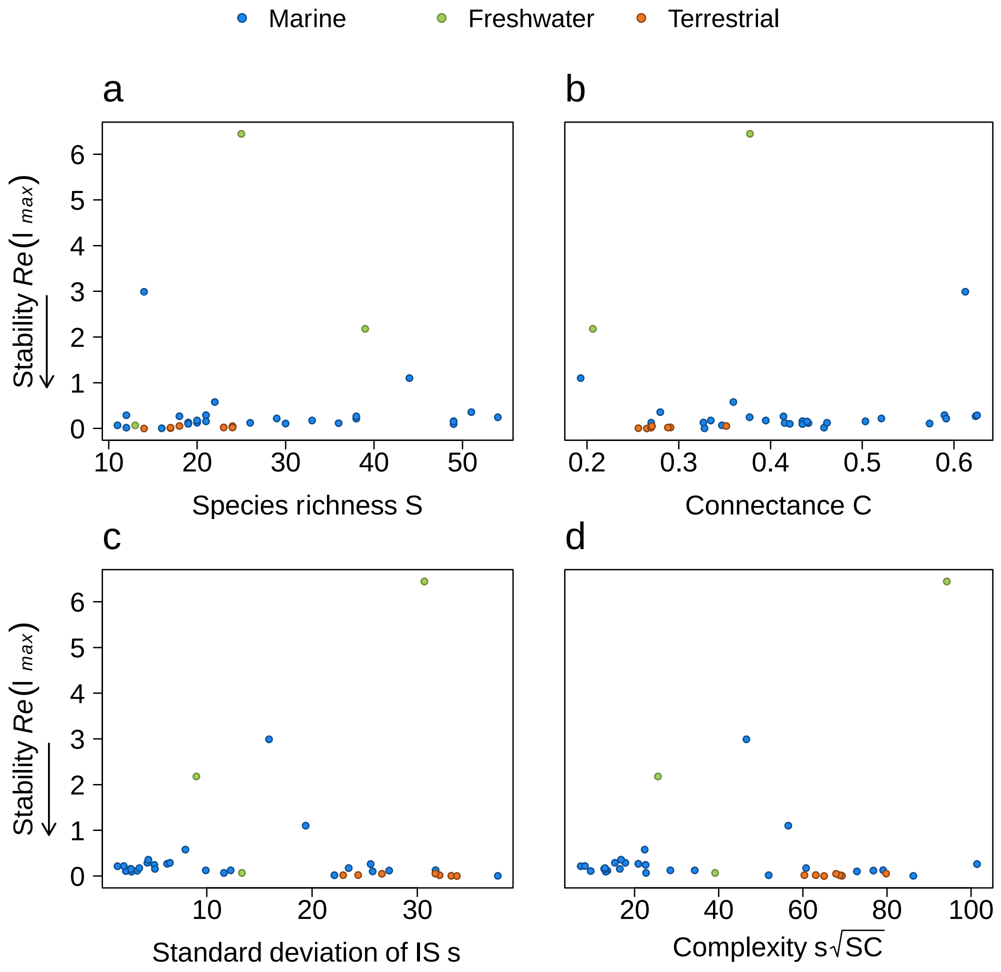

Supplementary Figure 1: Food web stability related to complexity parameters for the 37 best resolved food webs. (a) Number of species *S* (linear regression: $P = 0.89$, $R^2 = 10^{-4}$), (b) Connectance $C = L/S^2$ where *L* is the number of links ($P = 0.95$, $R^2 = 10^{-5}$), (c) Standard deviation of interaction strengths (IS) σ ($P = 0.49$, $R^2 = 0.01$), (d) May's complexity criterion $\sigma\sqrt{SC}$ ($P = 0.19$, $R^2 = 0.04$). Stability is measured as $Re(\lambda_{max})$, for marine (blue), freshwater (green) and terrestrial ecosystems (orange). Food webs with eigenvalues close to zero are the most stable. All quantities are dimensionless.

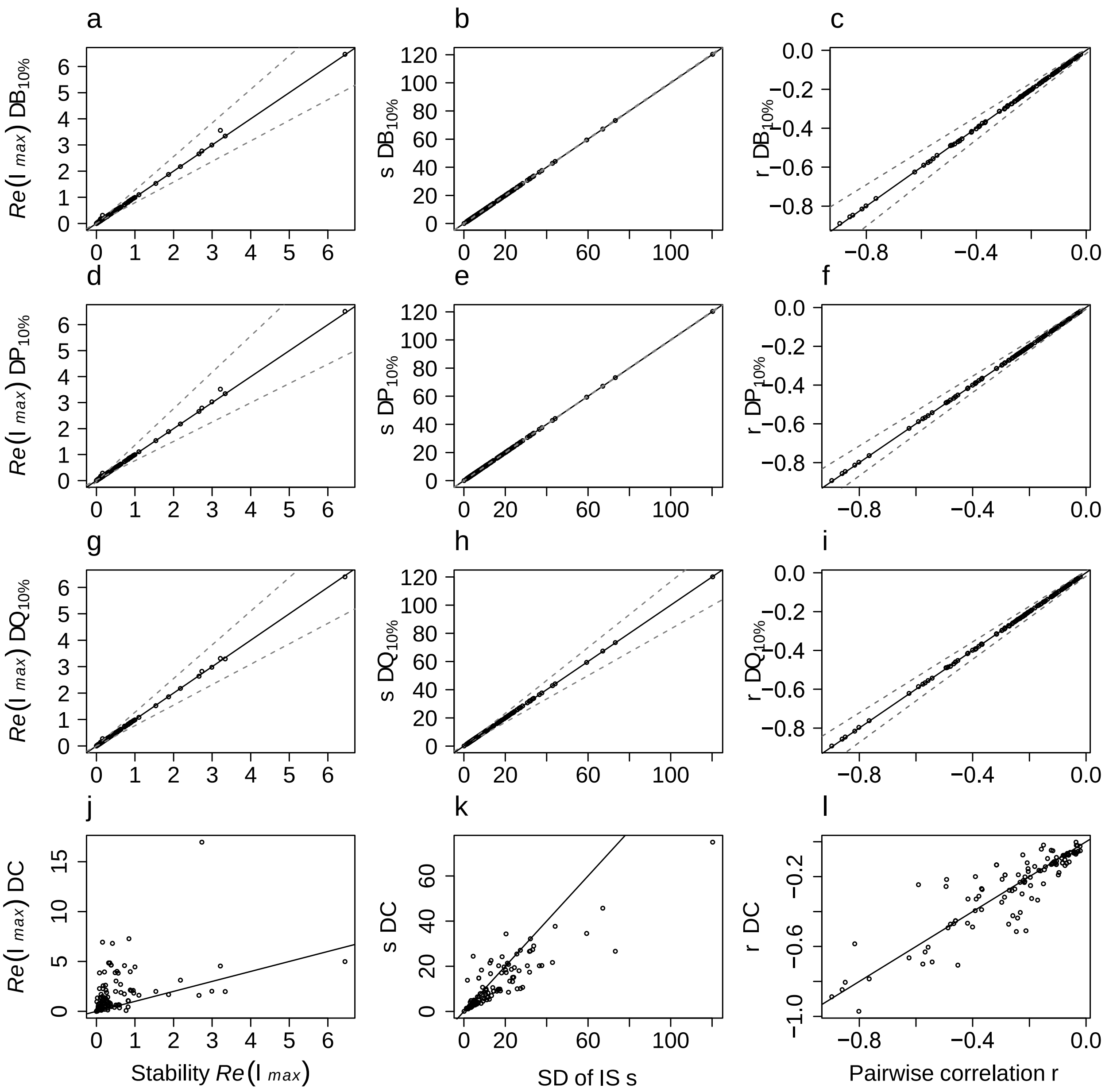

Supplementary Figure 2: Effect of input parameter variability on stability and descriptors of complexity. Output parameters: $Re(\lambda_{max})$ corresponds to stability (first column), σ is standard deviation of interaction strengths (IS) (second column) and ρ represents correlation between pairs of interactions (third column). Input parameters are biomass B (a-c), production/biomass ratio P/B (d-f), consumption/biomass ratio Q/B (g-i) and diet composition DC (j-l). Points represent the mean value of 1,000 replicates of the resampling procedure, dotted lines correspond to the 2.5% and 97.5% quantile regressions. Black lines correspond to 1:1 ratio between the original output parameter estimates (*x*-axis) and the resampled ones (*y*-axis).

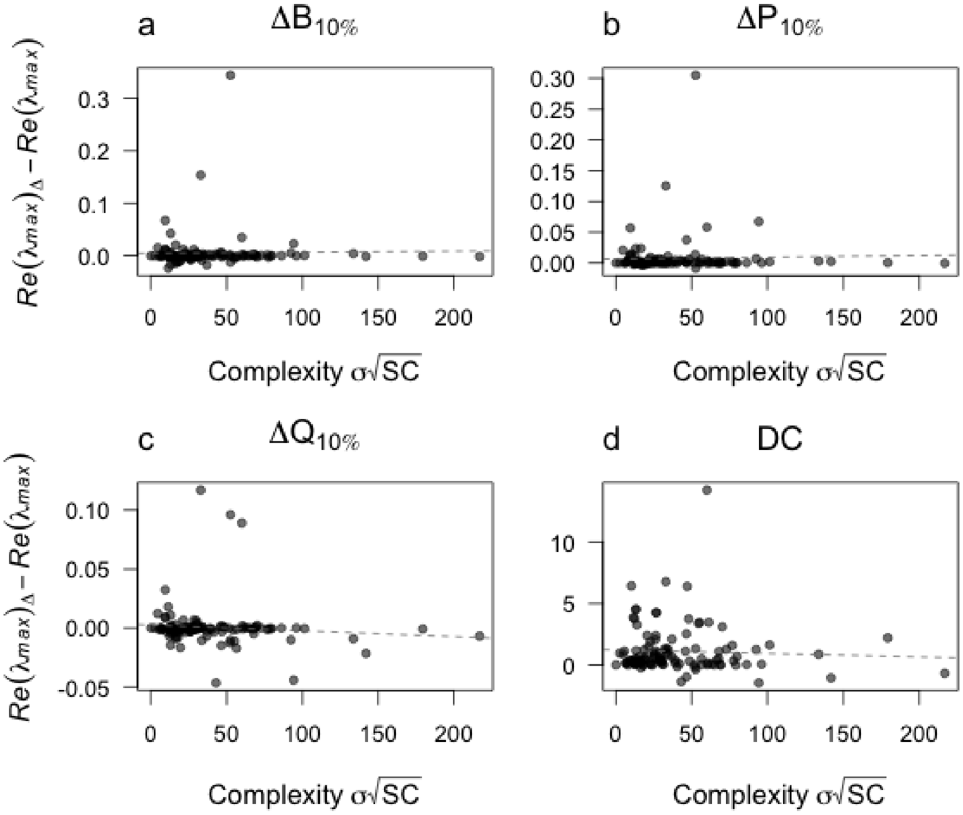

Supplementary Figure 3: Effect of complexity on the deviation between the stability of empirical or resampled community matrices. $Re(\lambda_{max})_{\Delta}$ and $Re(\lambda_{max})$ correspond to the stability of resampled and original community matrices respectively. Dotted lines illustrate the linear regression between complexity $\sigma\sqrt{SC}$ and $Re(\lambda_{max})_{\Delta} - Re(\lambda_{max})$. The resampled community matrices are drawn from the resampling of (a) species biomass *B* ($P = 0.82$, $R^2 = 10^{-4}$), (b) production/biomass ratio *P*/*B* ($P = 0.75$, $R^2 = 10^{-3}$) or (c) consumption/biomass ratio *Q*/*B* with a CV of 10% ($P = 0.32$, $R^2 = 10^{-2}$). (d) The community matrices are drawn from a diet composition matrix in which predators have no prey preferences ($P = 0.59$, $R^2 = 10^{-3}$).

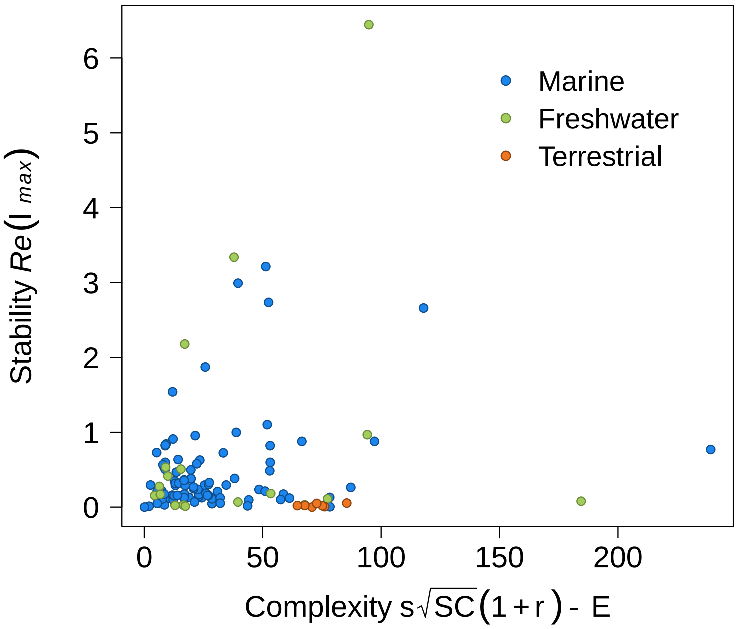

Supplementary Figure 4: Complexity-stability relationship in 116 food webs with Tang's complexity criterion. Complexity is defined as $\sigma\sqrt{SC}(1+\ \rho) - E)$, where E is the mean (including zeros) and ρ is the correlation between pairs of interactions. Stability is measured as *Re(*λ_{max}*)*, for marine (blue), freshwater (green) and terrestrial ecosystems (orange). Linear regression: P = 0.02, R^2 = 0.053. Food webs with eigenvalues close to zero are the most stable. All quantities are dimensionless.

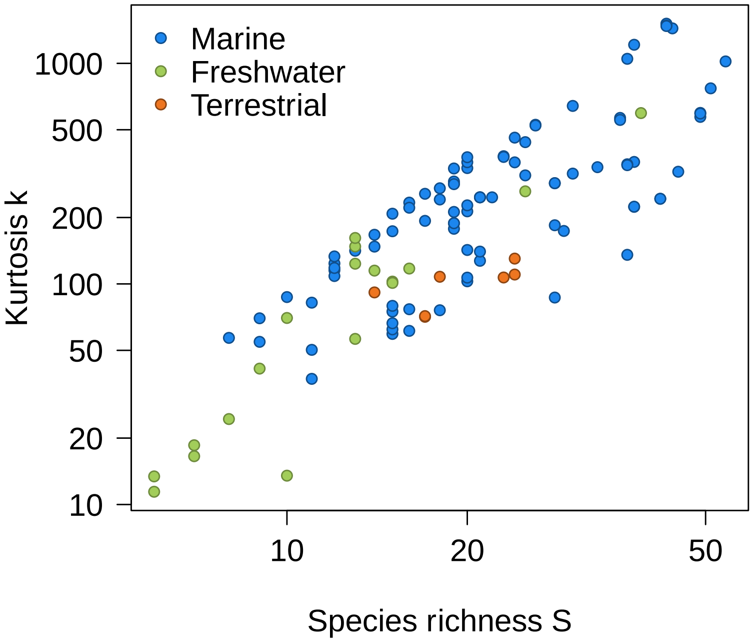

Supplementary Figure 5. Relationship between species richness and the shape of interaction strength distribution. The kurtosis κ is an index of the peakedness of the interaction strength distribution, for a normal distribution $\kappa = 0$. Kurtosis increases linearly with species richness on a logarithmic scale (linear regression: $P < 10^{-15}$, $R^2 = 0.71$). Marine, freshwater and terrestrial food webs are illustrated in blue, green and orange respectively. All quantities are dimensionless.

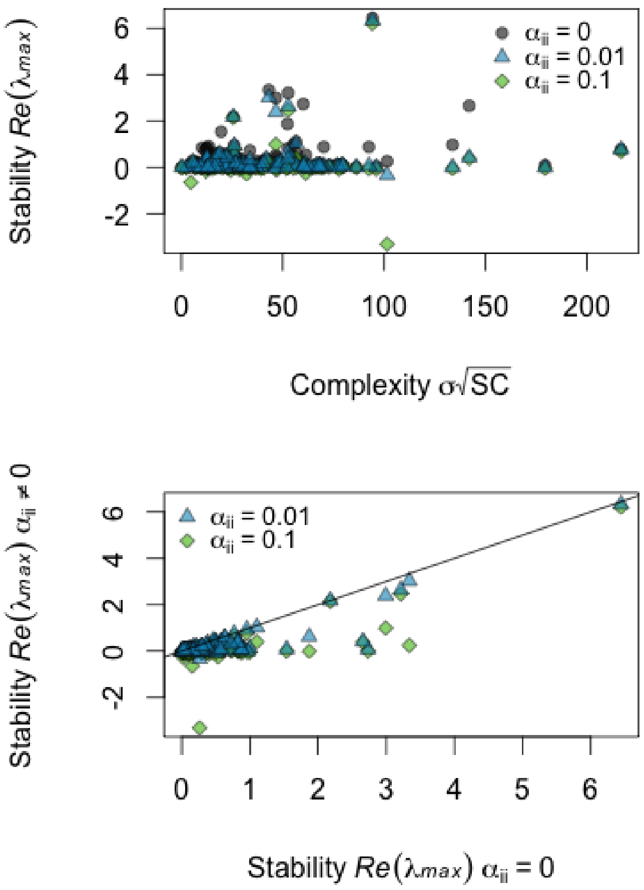

Supplementary Figure 6. Effect of diagonal elements on the complexity-stability relationship. (a) Complexity-stability relationship in 116 food webs with no intraspecific interactions (black circles, P = 0.02, R^2 = 0.04), with α_{ii} = 0.01 (blue triangles, P = 0.1, R^2 = 0.01) or α_{ii} = 0.1 (green diamonds, P = 0.34, R^2 = 10^{-3}). Complexity is defined as $\sigma\sqrt{SC}$ and stability is measured as $Re(\lambda_{max})$. (b) Relationships between stability of food web matrices with zero (x-axis) or non-zero diagonal elements (y-axis, α_{ii} = 0.01 or 0.1). The black line corresponds to 1:1 ratio between the x-axis and y-axis.

Supplementary Table

Supplementary Table 1. Dataset information. List of the 116 Ecopath models with references, habitat types, number of species *S*, connectance *C*, standard deviation of interaction strengths *σ*, and resolution index *RI.*

Model Name	Reference	Habitat	*S*	*C*	*σ*	*RI*
Alaska Prince William Sound OM	Dalsgaard et al. 1997[1]	marine	18	0.33	12.85	0.62
Alto Golfo De California	Morales-Zárate et al. 2004[2]	marine	28	0.53	2.99	0.63
Antarctica Weddel Sea	Jarre-Teichmann et al. 1997[3]	marine	19	0.25	4.38	0.57
Arctic islands, Alert	Legagneux et al.[4]	terrestrial	17	0.26	33.22	0.86
Arctic islands, Bylot	Legagneux et al.[4]	terrestrial	18	0.35	31.71	0.85
Arctic islands, Herschel	Legagneux et al.[4]	terrestrial	24	0.27	26.63	0.85
Arctic islands, Nenetsky	Legagneux et al.[4]	terrestrial	24	0.29	22.95	0.83
Arctic islands, Svalbard	Legagneux et al.[4]	terrestrial	14	0.27	33.76	0.76
Arctic islands, Yamal	Legagneux et al.[4]	terrestrial	23	0.29	24.38	0.84
Arctic islands, Zackenberg	Legagneux et al.[4]	terrestrial	17	0.27	32.11	0.86
Bali Strait	Buchary et al. 2002[5]	marine	13	0.58	16.7	0.51
Bay Of Biscay 1998	Ainsworth et al. 2001[6]	marine	36	0.62	2.58	0.42
Bay Of Somme	Rybarczyk et al. 2003[7]	marine	8	0.41	120.28	0.33
Black Sea	Orek 2000[8]	marine	9	0.42	21.52	0.43
Brunei Darussalam, China Sea	Silvestre et al. 1993[9]	marine	12	0.61	20.1	0.45
Campeche Bank, Golf of Mexico	Vega-Cendejas et al. 1993[10]	marine	18	0.51	7.09	0.6
Campeche Sound	Zetina-Rejon M.J. et al. 2003[11]	marine	24	0.52	5.36	0.61
Cape Verde	Stobberup et al. 2004[12]	marine	24	0.5	13.91	0.61
Caribbean	Melgo et al. 2009[13]	marine	28	0.31	4.56	0.68
Celestun	Chavez et al. 1993[14]	marine	15	0.51	4.45	0.6
Central Atlantic 50s	Vasconcellos & Watson 2004[15]	marine	37	0.29	4.01	0.56
Central Chile 1992	Neira et al. 2004[16]	marine	20	0.27	12.27	0.93
Central Gulf Of California	Arreguin-Sanchez et al. 2002[17]	marine	25	0.37	5.88	0.63
Central Pacific, sharks	Kitchell et al. 2002[18]	marine	21	0.59	4.35	0.8
Chesapeake Present	Christensen et al. 2009[19]	marine	44	0.19	19.4	0.79
Darwin Harbour, Australia	Martin 2005[20]	marine	20	0.49	6.57	0.5
Eastern Scotian Shelf 90s	Bundy 2004[21]	marine	38	0.59	1.52	0.72
Eastern Tropical Pacific	Olson & Walters 2003[22]	marine	38	0.41	25.56	0.81

Model Name	Reference	Habitat	S	C	σ	RI
Etang de Thau, France	Palomares et al. 1993[23]	freshwater	10	0.78	1.66	0.52
Gambia 1986	Mendy 2003[24]	marine	21	0.39	10.53	0.63
Gambia 1992	Mendy 2004[25]	marine	21	0.39	10.53	0.63
Gambia 1995	Mendy 2004[25]	marine	21	0.38	10.66	0.63
Gironde Estuary, France	Lobry 2004[26]	marine	16	0.39	7.83	0.56
Golfo Dulce, Costa Rica	Wolff et al. 1996[27]	marine	20	0.54	9.2	0.6
Guinee 1985	Guénette & Diallo 2004[28]	marine	43	0.44	5.9	0.66
Guinee 1998	Guénette & Diallo 2004[28]	marine	43	0.43	5.94	0.66
Guinee Bissau 1991	Amorim et al. 2003[29]	marine	30	0.43	7.23	0.57
Gulf of Salamanca, Upwelling	Duarte & Garcia 2004[30]	marine	17	0.59	8.32	0.54
High Barents Sea AllJuvs1990	Blanchard et al. 2002[31]	marine	15	0.49	4.82	0.54
High Barents Sea Final 1990	Blanchard et al. 2002[31]	marine	38	0.35	3.45	0.66
Huizache Caimanero, Mexico	Zetina-Rejón et al. 2004[32]	marine	25	0.51	2.88	0.64
Iceland Fisheries	Buchary 2001[33]	marine	20	0.54	2.05	0.69
Jalisco y Colima	Galván-Piña 2005[34]	marine	36	0.44	3.4	0.7
Kuala Terengganu, Malaysia	Liew & Chan 1987[35]	marine	12	0.61	20.42	0.4
Kuosheng Bay, Taiwan	Lin et al. 2004[36]	marine	16	0.28	18.45	0.49
Lagoon of Venice	Carrer & Opitz 1999[37]	marine	15	0.32	1.17	0.48
Laguna De Bay, Philippines, 1950	Delos Reyes 1995[38]	marine	20	0.34	23.48	0.73
Laguna De Bay, Philippines, 1980	Delos Reyes & Martens 1993[39]	marine	16	0.33	37.65	0.76
Laguna De Bay, Philippines, 1990	Delos Reyes 1995[38]	marine	19	0.33	31.73	0.73
Lake Aydat, France	Reyes-Marchant et al. 1993[40]	freshwater	10	0.66	16.77	0.49
Lake Chad, Africa	Palomares et al. 1993[41]	freshwater	14	0.59	16.35	0.55
Lake George, Uganda	Moreau et al. 1993[42]	freshwater	13	0.66	13.34	0.72
Lake Kariba, Africa	Machena et al. 1993[43]	freshwater	9	0.35	18.2	0.67
Lake Kinneret, Israel	Walline et al. 1993[44]	freshwater	13	0.39	59.34	0.52
Lake Malawi 2, Africa	Degnbol 1993[45]	freshwater	8	0.59	44.1	0.63
Lake Malawi, Africa	Nsiku 1999[46]	freshwater	25	0.38	30.67	0.7
Lake Tanganyka, Africa, 1975	Moreau et al. 1993a[47]	freshwater	6	0.78	28.38	0.5
Lake Tanganyka, Africa, 1981	Moreau et al. 1993a[47]	freshwater	6	0.78	21.5	0.5
Lake Turkana, Kenya, 1973	Kolding 1993[48]	freshwater	7	0.49	13.1	0.57
LakeTurkana, Kenya, 1987	Kolding 1993[48]	freshwater	7	0.49	12.57	0.57
Lake Victoria, Africa, 1971	Moreau et al. 1993[49]	freshwater	15	0.68	11.62	0.62

**Model Name**	**Reference**	**Habitat**	***S***	***C***	σ	***RI***
Lake Victoria, Africa, 1985	Moreau et al. 1993[49]	freshwater	15	0.71	11.32	0.62
Looe Key, Florida, USA	Venier & Pauly 1997[50]	marine	19	0.53	10.83	0.51
Low Barents Sea 1995	Blanchard et al. 2002[31]	marine	38	0.35	3.42	0.69
Low Barents Sea Juvs 1995	Blanchard et al. 2002[31]	marine	15	0.48	4.97	0.54
Mandinga Lagoon, Mexico	De La Cruz-Aguero 1993[51]	marine	19	0.33	10.37	0.65
Maputo Bay, Mozambique	De Paula et al. 1993[52]	marine	9	0.52	42.8	0.5
Mid Atlantic Bight	Okey 2001[53]	marine	54	0.38	5.01	0.7
Monterey Bay, California	Olivieri et al. 1993[54]	marine	15	0.33	23.54	0.36
Moorea Barrier reef	Arias-González et al. 1997[55]	marine	45	0.34	8.47	0.54
Moorea Fringing reef	Arias-González et al. 1997[55]	marine	42	0.33	7.18	0.56
Morocco 1984	Stanford et al. 2001[56]	marine	37	0.44	3.91	0.52
Newfoundland Grand Banks 1900	Heymans & Pitcher 2002[57]	marine	49	0.43	2.76	0.72
Newfoundland Grand Banks mid-1980s	Bundy 2001[58]	marine	30	0.57	2.3	0.76
Newfoundland Grand Banks mid-1980s	Heymans & Pitcher 2002[59]	marine	49	0.43	2.85	0.76
Newfounland Grand Banks mid-1990s	Heymans & Pitcher 2002[59]	marine	49	0.44	2.81	0.76
North Atlantic 1950s	Vasconcellos & Watson 2004[15]	marine	37	0.3	5.06	0.59
North Atlantic 1990s	Vasconcellos & Watson 2004[15]	marine	37	0.3	5.07	0.59
Northwest Africa	Morissette et al. 2010[60]	marine	26	0.28	21.03	0.69
Orbetello Lagoon	Brando et al. 2005[61]	marine	11	0.35	11.63	0.73
Pallude Della Rosa Lagoon Venice	Carreer et al. 2000[62]	marine	11	0.25	0.07	0.4
Patos Lagoon Estuary	Betito & Castro 1995[63]	marine	23	0.51	17.6	0.65
Peruvian upwelling system 1950s	Jarre-Teichmann 1998[64]	marine	19	0.42	27.33	0.7
Peruvian upwelling system 1960s	Jarre-Teichmann 1998[64]	marine	19	0.42	25.76	0.7
Ria Formosa	Gamito & Erzini 2005[65]	freshwater	13	0.62	24.05	0.58
Sakumo Lagoon, Ghana	Pauly 2002[66]	marine	12	0.28	36.46	0.65
San Pedro Bay, Leyte, Philippines	Campos 2003[67]	marine	15	0.63	9.26	0.48
Seine Estuary	Rybarczyk & Elkaïm 2003[68]	marine	14	0.41	20.34	0.64
Sene-Gambia	Samb & Mendy 2004[69]	marine	16	0.41	27.27	0.56
Sierra Leone 1964	Heymans & Vakily 2004[70]	marine	43	0.4	6.91	0.61
Sierra Leone 1978	Heymans & Vakily 2004[70]	marine	43	0.41	6.9	0.61

Model Name	Reference	Habitat	*S*	*C*	σ	*RI*
Sierra Leone 1990	Heymans & Vakily 2004[70]	marine	43	0.41	6.9	0.61
Sonda Campeche	Manickchand-Heileman et al. 1998[71]	marine	18	0.62	6.23	0.7
South Pacific, marine mammals	Morissette (unpublished data)	marine	42	0.33	7.18	0.55
Southern Brazil	Vasconcellos & Gasalla 2001[72]	marine	12	0.46	22.12	0.73
Southern Gulf St Lawrence 1980	Savenkoff et al. 2004[73]	marine	29	0.52	2.11	0.7
Southwest Coast Of India	Vivekanandan et al. 2003[74]	marine	10	0.54	17.41	0.4
SriLanka Lake Prakrama Samudra	Moreau et al. 2001[75]	freshwater	16	0.38	73.22	0.63
Strait Of Georgia	Martell et al. 2002[76]	marine	26	0.46	9.9	0.73
Subantartic Plateau New Zealand	Bradford-Grieve et al. 2003[77]	marine	17	0.26	67.13	0.54
Tamiahua Lagoon, Golf of Mexico	Abarca-Arenas & Valero-Pacheco 1993[78]	marine	12	0.62	6.5	0.7
Tampa Bay	Walters et al. 2005[79]	marine	51	0.28	4.45	0.86
Tampamachoco Lagoon, Mexico	Rosado-Solorzano & Guzman del Proo 1998[80]	marine	22	0.36	7.97	0.7
Terminos Lagoon, Gulf of Mexico	Manickchand-Heileman et al. 1998[81]	marine	19	0.65	3.94	0.6
Terminos Lagoon, seagrass	Rivera-Arriaga et al. 2003 [82]	marine	15	0.52	11.04	0.56
UK Virgin Islands, Caribbean	Opitz 1993[83]	marine	20	0.55	10.19	0.45
Upper Parana River Floodplain	Angelini & Agostinho 2005[84]	freshwater	39	0.21	9.01	0.82
Veli Lake, India	Aravindan 1993[85]	freshwater	13	0.45	32.13	0.58
West Coast of Greenland	Pedersen & Zeller 2001[86]	marine	21	0.5	5.08	0.74
West Coast of Sabah	Garces et al. 2003[87]	marine	28	0.48	14.32	0.6
West Coast of Sarawak	Garces et al. 2003[87]	marine	28	0.48	14.31	0.6
West Coast of Vancouver Island	Martell 2002[88]	marine	14	0.61	15.91	0.83
West Greenland, Shrimp Pound	Pedersen 1994[89]	marine	11	0.48	2.46	0.59
Western Bering Sea	Aydin et al. 2002[90]	marine	33	0.39	3.59	0.72
Western Gulf of Mexico	Arreguin-Sanchez et al. 1993[91]	marine	23	0.45	5.85	0.65
Yucatan shelf, Gulf of Mexico	Arreguin-Sanchez et al. 1993[92]	marine	20	0.52	10.41	0.63

Supplementary References

1. Dalsgaard, J., Pauly, D. & Okey, T. A. Preliminary mass-balance model of Prince William Sound, Alaka, for the pre-spill period, 1980-1989. *Fish. Cent. Res. Reports* **5,** 1–33 (1997).

2. Morales-Zárate, M. V., Arreguín-Sánchez, F., López-Martínez, J. & Lluch-Cota, S. E. Ecosystem trophic structure and energy flux in the Northern Gulf of California, México. *Ecol. Modell.* **174,** 331–345 (2004).

3. Jarre-teichmann, A. A. *et al.* in *Antarct. communities species, Struct. Surviv.* (Valencia, J., Walton, D. W. H. & Battaglia, B.) 118–134 (Cambridge University Press, 1997).

4. Legagneux, P. *et al.* Arctic ecosystem structure and functioning shaped by climate and herbivore body size. *Nat. Clim. Chang.* **4,** 379–383 (2014).

5. Buchary, E. A., Alder, J., Subhat, N. & Tonny, W. in *Use Ecosyst. Model. to Investig. Multispecies Manag. Strateg. Capture Fish.* (Pitcher, T. J. & Cochrane, K.) 24–32 (Fisheries Centre Reports 10(2), 2002).

6. Ainsworth, C., Feriss, B., Leblond, E. & Guenette, S. in *Fish. impacts North Atl. Ecosyst. Model. Anal.* (Guénette, S., Christensen, V. & Pauly, D.) 271–313 (Fisheries Centre Research Reports 9(4), 2001).

7. Rybarczyk, H., Elkaim, B., Ochs, L. & Loquet, N. Analysis of the trophic network of a macrotidal ecosystem: the Bay of Somme (Eastern Channel). *Estuar. Coast. Shelf Sci.* **58,** 405–421 (2003).

8. Orek, H. An application of mass balance Ecopath model to the trophic structure in the Black Sea after anchovy collapse. *Ms. In Marine Science, Middle East Technical University, Turkey* (2000).

9. Silvestre G, Selvanathan S, S. A. in *Trophic Model. Aquat. Ecosyst.* (Christensen, V. & Pauly, D.) 300–306 (ICLARM. Conf. Proc., Manila, 1993).

10. Vega-Cendejas, M. in *Trophic Model. Aquat. Ecosyst.* (Christensen, V. & Pauly, D.) 206–213 (ICLARM. Conf. Proc., Manila, 1993).

11. Zetina-Rejón M.J., Arreguın-Sánchez F., Uribe-Martinez J. *Memorias del III Foro de Camarón del Golfo de México y Mar Caribe* (Wakida Kusunoki A.T., Solana-Sansores R., Uribe-Martinez J.,) 49-57 (Secretaría de Agricultura, Ganadería, Desarrollo Rural, Pesca y Alimentación; Instituto Nacional de la Pesca, 2003)

12. Stobberup, K., Ramos, V. & Coelho, M. in *West African Mar. Ecosyst. Model. Fish. Impacts* (Palomares M.L.D., Pauly D.) 39–56 (Fisheries Centre Research Reports 12(7), 2004).

13. Melgo J.L., Morisette L., Kaschner K., Gerber L. in *Modelling the trophic role of marine mammals in tropical areas: data requirements, uncertainty, and validation* (Morissette L., Melgo J.L., Kaschner K., Gerber L.) 53-120 (Fisheries Centre Research Reports 17(2), 2009).

14. Chavez, E., Garduno, M. & Sanchez, F. in *Trophic Model. Aquat. Ecosyst.* (Christensen, V. & Pauly, D.) 186–192 (ICLARM. Conf. Proc., Manila, 1993).

15. Vasconcellos, M. & Watson, R. in *West African Marine Ecosystems: Models and Fisheries Impact* (Pauly, D. & Palomares, M.), 171–204 (Fisheries Centre Research Reports 12(7), 2004).

16. Neira, S., Arancibia, H. & Cubillos, L. Comparative analysis of trophic structure of commercial fishery species off Central Chile in 1992 and 1998. *Ecological Modelling* **172**, 233–248 (2004).

17. Arreguin-Sanchez, F., Arcos, E. & Chavez, E. Flows of biomass and structure in an exploited benthic ecosystem in the Gulf of California, Mexico. *Ecological Modelling* **156**, 167–183 (2002).

18. Kitchell, J. F., Essington, T. E., Boggs, C. H., Schindler, D. E. & Walters, C. J. The Role of Sharks and Longline Fisheries in a Pelagic Ecosystem of the Central Pacific. *Ecosystems* 5**,** 202–216 (2002).

19. Christensen V., Beattie A., Buchanan C., Hongguang M., Martell S.J.D., Latour R.J., Preikshot D., Sigrist M.B., Uphoff J.H., Walters C.J., Wood R.J., Townsend H. *Fisheries Ecosystem Model of the Chesapeake Bay: Methodology, Parameterization, and Model Exploration*, 1-146 (NOAA Technical Memorandum, 2009).

20. Martin, J. *The distribution, abundance and trophic ecology of the fishes of Darwin Harbour mangrove habitats.* Ph.D. thesis, Charles Darwin Univeristy, Darwin, Australia (2005).

21. Bundy, A. Mass balance models of the eastern Scotian Shelf before and after the cod collapse and other ecosystem changes, 1-193 (Canadian Technical Reports of Fisheries and Aquatic Sciences 2520, 2004).

22. Olson, R. & Watters, G. A model of the pelagic ecosystem in the eastern tropical pacific ocean. *Inter-AmericanTropical Tuna Commission Bulletin* **22**, 219 (2003).

23. Palomares, M., Horton, K. & Moreau, J. in *Trophic Model. Aquat. Ecosyst.* (Christensen, V. & Pauly, D.), 224–229 (ICLARM. Conf. Proc., Manila, 1993).

24. Mendy, A. in *West African Marine Ecosystems: Models and Fisheries Impact* (Pauly, D. & Palomares, M.), 81–88 (Fisheries Centre Research Reports 12(7), 2004).

25. Mendy, A. in *West African Marine Ecosystems: Models and Fisheries Impact* (Pauly, D. & Palomares, M.), 89–94 (Fisheries Centre Research Reports 12(7), 2004).

26. Lobry, J. *Quel référentiel de fonctionnement pour les écosystèmes estuariens?* Ph.D. thesis, University of Bordeaux, France. (2004).

27. Wolff, M., Hartmann, H. & Koch, V. A pilot trophic model for Golfo Dulce, a fjord-like tropical embayment, Costa Rica. *Revista de Biologia Tropical* **44**, 215–231 (1996).

28. Guénette, S. & Diallo, I. in *West African Marine Ecosystems: Models and Fisheries Impact* (Pauly, D. & Palomares, M.), 124–159 (Fisheries Centre Research Reports 12(7), 2004).

29. Amorim, P., Duarte, G., Guerra, M., Morato, T. & Stobberup, K. in *West African Marine Ecosystems: Models and Fisheries Impact* (Pauly, D. & Palomares, M.), 95–112 (Fisheries Centre Research Reports 12(7), 2004).

30. Duarte, L. O. & Garcia, C. B. Trophic role of small pelagic fishes in a tropical upwelling ecosystem. *Ecological Modelling* **172**, 323–338 (2004).

31. Blanchard, J.L., Pinnegar, J.K. & Mackinson, S. Exploring marine mammal-fishery interactions using 'Ecopath with Ecosim': modelling the Barents Sea ecosystem. Science Series Technical Report 117 (2002).

32. Zetina-Rejon, J. M., Arreguin-Sanchez, F. & Chavez, E. A. Trophic structure and flows of energy in the Huizache-Caimanero lagoon complex on the Pacific coast of Mexico. *Estuar. Coast. Shelf Sci.* **57,** 803–815 (2003).

33. Mendy A. N. Trophic modelling as a tool to evaluate and manage Iceland's multispecies fisheries (Fisheries Training Programme, 1999).

34. Galván Piña V.H. Impacto de la pesca en la estructura, funcion y productividad del ecosistema de la plataforma continental de las costas de Jalisco y Colima, Mexico. Ph.D. thesis, Instituto Politecnico National, Mexico (2005).

35. Liew, H. & Chan, E. *Ecopath model of a tropical shallow-water community in Malaysia*. Fisheries and Marine Science Centre, Universiti Pertanian Malaysia, Kuala Terengganu. (1987).

36. Lin, H., Shao, K. & Hwang, J. A trophic model for Kuosheng Bay in northern Taiwan. *Journal of Marine Science and Technology* **12**, 424–432 (2004).

37. Carrer, S. & Opitz, S. Trophic network model of a shallow water area in the northern part of the Lagoon of Venice. *Ecol. Modell.* **124,** 193–219 (1999).

38. Delos Reyes, M. *Geoecology of Laguna de Bay, Philippines: long-term alterations of a tropical-aquatic ecosystem 1820-1992*. Ph.D. thesis, University of Hamburg (1995).

39. Delos Reyes, M. & Martens, R. Geoecology of Laguna de Bay, Philippines I. Techno-commercial impact on the trophic level structure of the Laguna de Bay aquatic ecosystem 1968–1980. *Ecol. Modell.* **75/76,** 497–509 (1994).

40. Reyes-Marchant, P., Jamet, J., Lair, N., Taleb, H. & Palomares, M. in *Trophic Model. Aquat. Ecosyst.* (Christensen, V. & Pauly, D.), 95–102 (ICLARM. Conf. Proc., Manila, 1993).

41. Palomares M.L.D., Horton K., Moreau J. in *Trophic Model. Aquat. Ecosyst.* (Christensen, V. & Pauly, D.), 153-158 (ICLARM. Conf. Proc., Manila, 1993).

42. Moreau, J., Christensen, V. & Pauly, D. in *Trophic Model. Aquat. Ecosyst.* (Christensen, V. & Pauly, D.), 124–129 (ICLARM. Conf. Proc., Manila, 1993).

43. Machena, C., Kolding, J. & Sanyanga, R. A in *Trophic Model. Aquat. Ecosyst.* (Christensen, V. & Pauly, D.), 130–137 (ICLARM. Conf. Proc., Manila, 1993).

44. Walline, P., Pisanty, S., Gophen, M. & Berman., T. in *Trophic Model. Aquat. Ecosyst.* (Christensen, V. & Pauly, D.), 103–109 (ICLARM. Conf. Proc., Manila, 1993).

45. Degnbol, P. in *Trophic Model. Aquat. Ecosyst.* (Christensen, V. & Pauly, D.), 110–115 (ICLARM. Conf. Proc., Manila, 1993).

46. Nsiku E. in *Use Ecosyst. Model. to Investig. Multispecies Manag. Strateg. Capture Fish.* (Pitcher, T. J. & Cochrane, K.) 54–67 (Fisheries Centre reports 10(2), 2002).

47. Moreau, J., Nyakageni, B., Pearce, M. & Petit, P. in *Trophic Model. Aquat. Ecosyst.* (Christensen, V. & Pauly, D.), 138–143 (ICLARM. Conf. Proc., Manila, 1993).

48. Kolding, J. in *Trophic Model. Aquat. Ecosyst.* (Christensen, V. & Pauly, D.), 116–123 (ICLARM. Conf. Proc., Manila, 1993).

49. Moreau, J., Ligtvoet, W. & Palomares, M. in *Trophic Model. Aquat. Ecosyst.* (Christensen, V. & Pauly, D.), 144–152 (ICLARM. Conf. Proc., Manila, 1993).

50. Venier, J. & Pauly, D. in *Proc. 8th Int. Coral Reef Symp.* (Lessios H.A., Macintyre I.G.) 1:915–920 (Smithsonian Tropical Research Institute, 1997).

51. De La Cruz-Aguero, G. D. L. in *Trophic Model. Aquat. Ecosyst.* (Christensen, V. & Pauly, D.), 193–196 (ICLARM. Conf. Proc., Manila, 1993).

52. Silva, R., Sousa, M. & Caramelo, A. in *Trophic Model. Aquat. Ecosyst.* (Christensen, V. & Pauly, D.), 214–223 (ICLARM. Conf. Proc., Manila, 1993).

53. Okey, T. & Pugliese, R. in *Fish. Impacts North Atl. Ecosyst. Model. Anal.* (Guenette, S., Christensen, V. & Pauly, D.) 167–181 (Fisheries Centre Research Reports 9(4), 2001).

54. Olivieri, R., Cohen, A. & Chavez, F. in *Trophic Model. Aquat. Ecosyst.* (Christensen, V. & Pauly, D.), 315–322 (ICLARM. Conf. Proc., Manila, 1993).

55. Arias-Gonzalez, J., Delesalle, B., Salvat, B. & Galzin, R. Trophic functioning of the Tiahura reef sector, Moorea Island, French Polynesia. *Coral Reefs* **16**, 231–246 (1997).

56. Stanford R., Lunn K., Guénette S., Guénette S. in *Fish. Impacts North Atl. Ecosyst. Model. Anal.* (Guenette, S., Christensen, V. & Pauly, D.) 314–344 (Fisheries Centre Research Reports 9(4), 2001).

57. Heymans, S. & Pitcher, T. in *Ecosyst. Model. Newfoundl. time periods 1995, 1985, 1900 1450.* (Pitcher, T. J., Heymans, J. J. & Vasconcellos, M.) 44–71 (Fisheries Centre Research Reports 10(5), 2002).

58. Bundy A., in *Information supporting past and present ecosystem models of Northern British Columbia and the Newfoundland shelf* (Pitcher T., Vasconcellos M., Heymans S.J.J., Brignall C., Haggan N.),. 13-21 (Fisheries Centre Research Reports 10(1), 2002).

59. Heymans, S. & Pitcher, T. in *Ecosyst. Model. Newfoundl. time periods 1995, 1985, 1900 1450.* (Pitcher, T. J., Heymans, J. J. & Vasconcellos, M.) 5–43 (Fisheries Centre Research Reports 10(4), 2002).

60. Morissette, L., Kaschner, K. & Gerber, L. Ecosystem models clarify the trophic role of whales off Northwest Africa. *Mar. Ecol. Prog. Ser.* **404,** 289–302 (2010).

61. Brando V.E., Ceccarelli R., Libralato S., Ravagnan G. Assessment of environmental management effects in a shallow water basin using mass-balance models. *Ecol. Modell.* **172,** 213–232 (2004).

62. Carrer, S., Halling-Sørensen, B. & Bendoricchio, G. Modelling the fate of dioxins in a trophic network by coupling an ecotoxicological and an Ecopath model. *Ecol. Modell.* **126,** 201–223 (2000).

63. Betito, R. & Castro, L.A.B. Um modelo ECOPATH II do estuário da Lagoa dos Patos: uma avaliação preliminar. XI Encontro Brasileiro de Ictiologia, Campinas (1995)

64. Jarre-Teichmann A., Pauly D. in *Trophic Model. Aquat. Ecosyst.* (Christensen, V. & Pauly, D.), 307–314 (ICLARM. Conf. Proc., Manila, 1993).

65. Gamito, S. & Erzini, K. Trophic food web and ecosystem attributes of a water reservoir of the Ria Formosa (south Portugal). *Ecol. Modell.* **181,** 509–520 (2005).

66. Pauly, D. in *The Gulf of Guinea Large Marine Ecosystem*: *Environmental Forcing & Sustainable Development of Marine Resources* (McGlade, M., Cury, P., Korateng, K. & Hardman-Mountford), 289–296 (Elsevier Science, 2002).

67. Campos, W. in *Assessment, Manag. Futur. Dir. Coast. Fish. Asian Ctries.* (Silvestre, G. et al.) 353–364 (WorldFish Center Conference Proceedings 67, 2003).

68. Rybarczyk, H. & Elkaïm, B. An analysis of the trophic network of a macrotidal estuary: the Seine Estuary (Eastern Channel, Normandy, France). *Estuar. Coast. Shelf Sci.* **58,** 775–791 (2003).

69. Samb, B. & Mendy, A. in *West African Marine Ecosystems: Models and Fisheries Impact* (Pauly, D. & Palomares, M.), 57–70 (Fisheries Centre Research Reports 12(7), 2004).

70. Heymans, S. & Vakily, J. in *West African Marine Ecosystems: Models and Fisheries Impact* (Pauly, D. & Palomares, M.), 160–169 (Fisheries Centre Research Reports 12(7), 2004).

71. Manickchand-Heileman, S. A preliminary trophic model of the continental shelf, south-western Gulf of Mexico. *Estuar. Coast. Shelf Sci.* **46,** 885–899 (1998).

72. Vasconcellos, M. & Gasalla, M. a. Fisheries catches and the carrying capacity of marine ecosystems in southern Brazil. *Fish. Res.* **50,** 279–295 (2001).

73. Savenkoff, C. et al. Input data and parameter estimates for ecosystem models of the southern Gulf of St. Lawrence (mid-1980s and mid-1990s, 111p. (Canadian Technical Reports of Fisheries and Aquatic Sciences 2529, 2004).

74. Vivekanandan, E., Srinath, M., Pillai, V., Immanuel, S. & Kurup., K. in *Assessment, Manag. Futur. Dir. Coast. Fish. Asian Ctries.* (Silvestre, G. et al.) 281–298 (WorldFish Center Conference Proceedings 67, 2003).

75. Moreau J., Villanueva M.C., Amarasinghe U.S., Schiemer F. in *Reservoir and culture-based fisheries: Biology and Management* (De Silva S.S), 201-214 (ACIAR, Canberra, Australia, 2001).

76. Martell, S., Beattie, A., Walters, C. J., Nayar, T. & Briese., R. in *Use Ecosyst. Model. to Investig. Multispecies Manag. Strateg. Capture Fish.* (Pitcher, T. J. & Cochrane, K.) 16–23 (Fisheries Centre reports 10(2), 2002).

77. Bradford-Grieve, J. M. *et al.* Pilot trophic model for subantarctic water over the Southern Plateau, New Zealand: a low biomass, high transfer efficiency system. *J. Exp. Mar. Bio. Ecol.* **289,** 223–262 (2003).

78. Abarca-Arenas, L. & Valero-Pacheco, E. in *Trophic Model. Aquat. Ecosyst.* (Christensen, V. & Pauly, D.), 181–185 (ICLARM. Conf. Proc., Manila, 1993).

79. Walters, C. J., Christensen, V., Martell, S. & Kitchell, J. F. Possible ecosystem impacts of applying MSY policies from single-species assessment. *ICES J. Mar. Sci.* **62,** 558–568 (2005).

80. Rosado-Solórzano, R. & Próo, S. G. del. Preliminary trophic structure model for Tampamachoco lagoon, Veracruz, Mexico. *Ecol. Modell.* **109,** 141–154 (1998).

81. Manickchand-Heileman, S., Arreguin-Sanchez, F., Lara-Dominguez, a. & Soto, L. A. Energy flow and network analysis of Terminos Lagoon, SW Gulf of Mexico. *J. Fish Biol.* **53,** 179–197 (1998).

82. Rivera-Arriaga, E., Lara-Dominguez, A. L., Villalobos-Zapata, G. & Yanez-Arancibia, A. in *From Mexico to Brazil: Central Atlantic Fisheries Catch Trends and Ecosystem Models* (Zeller D., Booth S., Pauly D.), 245-254 (Fisheries Centre Research Reports 11(6), 2003).

83. Opitz S. in *Trophic Model. Aquat. Ecosyst.* (Christensen, V. & Pauly, D.), 259–267 (ICLARM. Conf. Proc., Manila, 1993).

84. Angelini, R. & Agostinho, A. Food web model of the Upper Parana River Floodplain: description and aggregation effects. *Ecol. Modell.* **181,** 109–121 (2005).

85. Aravindan, C. in *Trophic Model. Aquat. Ecosyst.* (Christensen, V. & Pauly, D.), 87–89 (ICLARM. Conf. Proc., Manila, 1993).

86. Pedersen, S. & Zeller, D. in *Fish. impacts North Atl. Ecosyst. Model. Anal.* (Guénette, S., Christensen, V. & Pauly, D.) 111–127 (Fisheries Centre Research Reports 9(4), 2001).

87. Garces, L. & Alias, M in *Assessment, Manag. Futur. Dir. Coast. Fish. Asian Ctries.* (Silvestre, G. et al.) 333–352 (WorldFish Center Conference Proceedings 67, 2003).

88. Martell, S. *Variation in pink shrimp populations off the west coast of Vancouver Island: oceanographic and trophic interactions*. Phd thesis, University of British Columbia (2002).

89. Pedersen, S. Multispecies interactions on the offshore West Greenland shrimp grounds. *Int. Counc. Explor. Sea C.* 26p. (1994).

90. Aydin, K., Lapko, V., Radchenko, V. & Livingston, P. A comparison of the eastern Bering and western Bering Sea shelf and slope ecosystems through the use of mass-balance food web models. NOAA Tech. Memo. NMFS-AFSC-130 (2002).

91. Arreguin-Sanchez, F., Valero-Pacheco E., Chavez E.A. in *Trophic Model. Aquat. Ecosyst.* (Christensen, V. & Pauly, D.), 197–205 (ICLARM. Conf. Proc., Manila, 1993).

92. Arreguin-Sanchez, F., Seijo J.C., Valero-Pacheco E. in *Trophic Model. Aquat. Ecosyst.* (Christensen, V. & Pauly, D.), 269–278 (ICLARM. Conf. Proc., Manila, 1993).
